# Case report: hypoglycemia secondary to methimazole-induced insulin autoimmune syndrome in young Taiwanese woman with Graves’ disease

**DOI:** 10.1097/MD.0000000000029337

**Published:** 2022-06-24

**Authors:** Hsuan-Yu Wu, I-Hua Chen, Mei-Yueh Lee

**Affiliations:** a School of Medicine, Kaohsiung Medical University, Kaohsiung, Taiwan; b Division of Endocrinology and Metabolism, Department of Internal Medicine, Kaohsiung Medical University, Kaohsiung, Taiwan; c Faculty of Medicine, College of Medicine, Kaohsiung Medical University, Kaohsiung, Taiwan.

**Keywords:** Graves’ disease, hypoglycemia, insulin, methimazole

## Abstract

**Rationale:**

Hypoglycemia is an emergent condition with many causes, including underlying diabetes mellitus either with the use of insulin or oral anti-diabetic medications for glucose control, and organ (heart, hepatic, or renal) failure. Insulin autoimmune syndrome (IAS) can also cause hypoglycemia, however it is relatively difficult to diagnose as it is rare clinically. Although uncommon, IAS can be life threatening in patients with persistent hypoglycemia.

**Patient concern:**

We report the case of a 27-year-old female with underlying Graves’ disease who was treated with methimazole (MTZ). After 6 weeks of treatment, she developed hypoglycemia symptoms accompanied by dizziness and cold sweating. We excluded underlying diabetes mellitus, the use of insulin or oral anti-diabetic medications, and organ failure.

**Diagnoses:**

Laboratory data showed elevated insulin and C-peptide levels. Therefore, insulinoma and IAS were suspected. Abdominal computed tomography and magnetic resonance imaging ruled out insulinoma, and MTZ-induced IAS was finally diagnosed.

**Interventions and outcomes:**

The hypoglycemia symptoms resolved after MTZ was switched to propylthiouracil, confirming the diagnosis of IAS.

**Lessons:**

This case emphasizes the significance of life-threatening MTZ-induced IAS. IAS should be suspected in patients who develop spontaneous hypoglycemia, especially in those with underlying Graves’ disease receiving MTZ who present with hyperinsulinism.

## 1 Introduction

Insulin autoimmune syndrome (IAS), also known as Hirata's disease or insulin autoimmune hypoglycemia, is a rare autoimmune disorder which can cause fasting and/or postprandial hypoglycemia with high serum concentrations of total immunoreactive insulin and the presence of insulin autoantibodies in those with no prior exposure to exogenous insulin.^[1–3]^ The disease was first reported in Japan by Hiram in 1970,^[4]^ and 400 cases have since been reported. It is less common in Western countries and more common in Eastern countries, especially in Japan.^[5]^ The peak onset of this condition is between 60 and 69 years of age.^[6]^ It is the third leading cause of hypoglycemia in Japan after insulinoma and extra-pancreatic neoplasm.^[7]^ However, IAS is difficult to diagnose in patients with new-onset hypoglycemia because it is usually obscured by causes such as sepsis, alcohol intoxication, malnutrition, insulin, and drug overdose. Various structural problems including adrenal insufficiency, excess insulin production from insulinoma, and islet hyperplasia can also cause hypoglycemia.^[8]^ Although the causes of IAS are unclear, the use of sulfhydryl group-containing medications such as methimazole (MTZ) and the HLA-DRB1∗0406 genotype have been reported to be predisposing factors.^[9,10]^ As relatively few cases have been reported in Taiwan, herein we share our experience of a case of MTZ-induced IAS to raise awareness of this potentially life-threatening disease.

## 2 Case report

A 27-year-old female with underlying Graves’ disease presented with cold sweating and dizziness after taking the anti-thyroid drug MTZ (10 mg twice daily). As a ward nurse of a local hospital, she routinely checked her finger sugar when symptoms occurred, and hypoglycemia was noted with a blood glucose level as low as 42 mg/dL. She then visited our outpatient department where she reported that the hypoglycemia events had occurred for 6 weeks after she started MTZ treatment for Graves’ disease. The symptoms were possibly aggravated after meals. She denied taking any form of anti-diabetic medications, however she reported a family history of diabetes mellitus. Thyroid function showed over-correction of hyperthyroidism with elevated thyroid stimulating hormone (TSH), low free thyroxine (free T4), and positive TSH receptor antibodies (Ab-TSH R) (Table 1). Thus, the dose of MTZ was tapered to 5 mg twice daily. Glycated hemoglobin (HbA1c) was checked to rule out diabetes mellitus, and liver and renal function tests excluded organ failure-induced hypoglycemia. A 50-g oral glucose tolerance test showed severe postprandial hypoglycemia after 3 hours (Table 1). In addition, radioimmunoassays showed a normal cortisol level and elevated insulin level (Table 1). Admission was arranged for 72-hour fasting test and imaging surveys to rule out abdominal lesions such as insulinoma or adrenal hyperplasia.

**Table 1:** Laboratory data during first visit and admission.

Laboratory data	First visit	During admission	After discharged	Normal range
HbA1c (%)	5.1			4–6
Insulin (μIU/mL)	35.52	38.17	14.72	2–17
C-peptide (ng/mL)		2.71		1.77–4.68
Cortisol (μg/dL)	11.90	9.17		4.7–23.3 (8–10 am)
TSH (μIU/mL)	8.88			0.25–4
Free T4 (ng/dL)	<0.24			0.7–1.8
Ab-TSH R (U/L)	24.99			<1.5
Thyroglobulin Ab (IU/mL)	176			<40
Microsomal Ab (IU/mL)	117			<35
GPT (IU/L)	18			10–40
Uric acid (mg/dL)	7.0			2.6–8.0
BUN (mg/dL)		13.5		8–20
Creatinine (mg/dL)	0.69	0.68		0.44–1.03
Oral glucose tolerance test (OGTT)				
Glucose (AC) (mg/dL)	93	97		65–109
Glucose (120 min) (mg/dL)	86			<155
Glucose (180 min) (mg/dL)	33			<140

72-h fasting test	First day	Second day	
Glucose (mg/dL)	105 (06:03)85 (12:08)86 (17:17)	82 (00:13)74 (06:02)	65–109

Ab = antibody, Ab-TSH R = thyroid stimulating hormone receptor antibody, AC = ante-cibum (before meals), BUN = blood urea nitrogen, FT4 = thyroxine, free, GPT = glutamic pyruvic transaminase, HbA1c = glycated hemoglobin, TSH = thyroid stimulating hormone.

**Figure 1:**
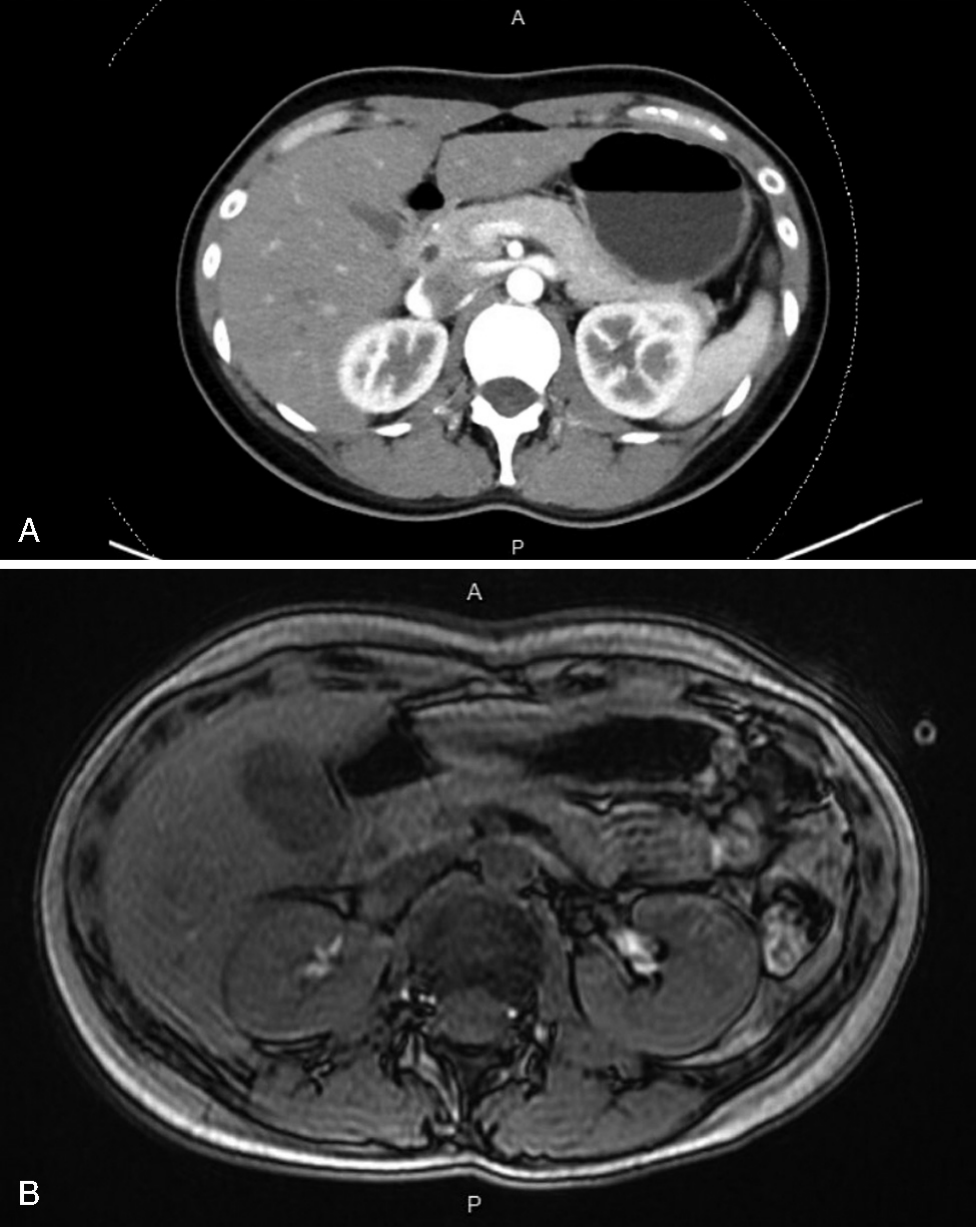
(A) Imaging result of abdominal computed tomography revealed no evidence of no evidence of pancreatic tumor nor insulinoma. (B) Imaging result of abdominal magnetic resonance imaging revealed no evidence of pancreatic lesion or insulinoma.

After admission, counter-regulatory hormones were checked. The cortisol level was within normal range, and with human growth hormone was mildly elevated, possibly indicating a physiologic reaction to hypoglycemia (Table 1). An elevated insulin level was highly suggestive of endogenous hyperinsulinemia, although C-peptide was within normal range (Table 1). Abdominal computed tomography (CT) and magnetic resonance imaging (MRI) showed no evidence of a pancreatic tumor or adrenal hyperplasia (Fig. 1). The 72-hour fasting test was started on the morning of day 3, but stopped on day 4 due to patient intolerance. We shifted MTZ to propylthiouracil on day 3 due to possible drug side effects causing IAS, after which no discomfort or hypoglycemia episodes were noted. Thus, after excluding other causes of hypoglycemia, we made our final diagnosis of MTZ-induced IAS. Her insulin level was rechecked during outpatient department follow-up visits, and found to be within normal range.

## 3 Discussion

This 27-year-old female had underlying Graves’ disease and presented with hypoglycemia and a high insulin but normal C peptide level after taking MTZ. After negative findings on both abdominal CT and MRI, we made the final diagnosis of IAS. IAS is seldom considered in patients presenting with hypoglycemia due to its rarity among most ethnicities, although it is the third leading cause of hypoglycemia in Japan.^[3,7]^ IAS is characterized by spontaneous hypoglycemia accompanied by hyperinsulinism and elevated insulin autoantibody tilters.^[1]^ IAS has been associated with autoimmune syndromes, including rheumatoid arthritis, polymyositis, systemic sclerosis, ankylosing spondylitis, and especially Graves’ disease.^[9]^Although uncommon clinically, IAS can lead to prolonged hypoglycemia and life-threatening consequences in some cases. Although it is not completely understood, IAS has a higher incidence in those receiving sulfhydryl group-containing medications such as MTZ, and in those with specific human leukocyte antigen (HLA) genotypes such as HLA-DRB1∗0406.^[10,11]^ A list of common drugs containing sulfhydryl groups is presented in Table 2.^[12]^ It has been suggested that the sulfhydryl group in these drugs may cleave the disulfide bond of insulin molecules, and bind to DRα-DRB1∗0406 on antigen-presenting cells with high affinity.^[13]^ This mechanism activates self-insulin T-helper cells, leading to the formation of insulin autoantibody complex which increases with time. This then leads to spontaneous hypoglycemia due to the surge of insulin release from the insulin autoantibody complex.^[8]^ Fortunately, although propylthiouracil has been associated with IAS in rare cases, this was not seen in our case when we switched MTZ to propylthiouracil.

**Table 2:** Insulin autoimmune syndrome (IAS) triggers.

Methimazole	Carbomazole
Propylthiouracil	Diltiazem
Alfa-mercaptopropionyl glycine	Alpha-lipoic acid
Glutathione	Methionine
Captopril	Hydralazine
Steroids	Penicillamine
Penicillin G	Imipenem
Pantoprazole	Clopidogrel

IAS clinically and biochemically mimics other causes of hypoglycemia, such as the iatrogenic administration of insulin, insulinoma, and type B insulin resistance syndrome.^[14]^ Thus, it is very important to differentiate these diseases. High C-peptide and insulin levels indicate that endogenous hypoglycemic agents such as sulfonylureas and insulinoma should be ruled out. Detailed medication history taking can be used to identify the use of sulfonylureas, and abdominal CT and/or MRI with 72-hour fasting test can be used to help exclude insulinoma. Most importantly, testing for insulin autoantibodies in non-diabetic adults with hyperinsulinemic hypoglycemia is recommended by the Endocrine Society as the first-line test in such patients.^[15]^ A limitation of this case is that insulin antibodies and HLA genotyping, especially for the HLA-DRB1∗0406 genotype, were not checked, as testing was not available at our center. If we could have conducted these tests, we may have diagnosed MTZ-induced IAS sooner. In our case, we made the diagnosis of IAS after excluding other causes of hypoglycemia, and due to the remission of hypoglycemia and insulin level after MTZ was withdrawn.

In approximately 80% of patients, IAS is self-limiting and quickly resolves after stopping the drug that has induced hypoglycemia. Therefore, the key to IAS treatment is to urgently identify the medication and not re-introduce it. On the other hand, patients with hypoglycemia are told to take small frequent meals with a reduced amount of carbohydrates to avoid sudden increases in plasma glucose leading to the over secretion of insulin.^[16]^ In some severe cases, acarbose, diazoxide, and octreotide, which play a role in decreasing or delaying absorption of carbohydrates in the intestine, may help relieve symptoms.^[17]^ If hypoglycemia persists, steroids as immunosuppressive therapy, azathioprine or 6-mercaptopurine combined with plasmapheresis can also serve as an alternative therapy. For refractory cases, rituximab, an anti-CD20 monoclonal antibody, can be tried to suppress an over-reactive immune system.^[18]^ In our case, the patient improved one day after MTZ had been withdrawn.

In conclusion, this case highlights the significance of life-threatening MTZ-induced IAS. In patients with spontaneous hypoglycemia, clinicians should always consider IAS, especially in those with underlying Graves’ disease who are receiving MTZ and present with hyperinsulinism.

## Author contributions

**Conceptualization:** Mei Yueh Lee.

**Data curation:** Chen I-Hua.

**Methodology:** Chen I-Hua.

**Project administration:** Mei Yueh Lee.

**Resources:** Mei Yueh Lee.

**Supervision:** Mei Yueh Lee.

**Writing – original draft:** Wu Hsuan-Yu.

**Writing – review & editing:** Mei Yueh Lee.
